# 1420. Descriptive Epidemiology of UTI Hospitalizations in the US, 2018

**DOI:** 10.1093/ofid/ofab466.1612

**Published:** 2021-12-04

**Authors:** Marya Zilberberg, Brian Nathanson, Kate Sulham

**Affiliations:** 1 EviMed Research Group, LLC, Goshen, MA; 2 OptiStatim, LLC, Longmeadow, MA; 3 Spero Therapeutics, Cambridge, MA

## Abstract

**Background:**

In parallel with an increase in antimicrobial resistance, urinary tract infections (UTI), one of the most common diagnoses among hospitalized patients in the US, have been on the rise. Though mostly emphasized as a hospital-acquired complication among patients with an indwelling catheter, quantification of the full contemporary burden of UTI-associated hospitalizations is limited.

**Methods:**

We conducted a cross-sectional multicenter study within the National Inpatient Sample (NIS) database, a 20-percent stratified sample of discharges from US community hospitals, from 2018, to explore characteristics of patients discharged with a UTI diagnosis. We divided UTI into mutually exclusive categories of complicated (cUTI), uncomplicated (uUTI), and catheter-associated (CAUTI). We applied survey methods to develop national estimates.

**Results:**

Among 2,837,385 discharges with a UTI code, 77.9% were uUTI, 17.6% cUTI, and 4.4% CAUTI. Compared to patients with uUTI (mean age 69.0 years), those with CAUTI and cUTI were older (70.1 and 69.7 years), but had same comorbidity burden (mean Charlson 4.3) as cUTI (4.3) and lower than CAUTI (4.6). Compared to other geographic regions, the Northeast had the lowest proportion of uUTI (74.6%) and highest of cUTI (20.8%) while the South had highest uUTI (80.2%) and lowest cUTI (15.7%). Over 60% of all UTI, regardless of type, were in large, and nearly ½ in urban teaching, institutions, and >80% came through the emergency department. Antimicrobial resistance codes were infrequent, but extended spectrum beta-lactamase organisms were more common in CAUTI (2.7%) and cUTI (2.1%) than in uUTI (1.6%). Among the 83.0% of discharges whose UTI was a secondary diagnosis, sepsis was the most common principal diagnosis, ranging from 17.7% in uUTI to 22.3% in cUTI. Although relatively low across the board, hospital mortality was lowest in cUTI (2.8%) and highest in uUTI (3.9%). Discharges to a chronic care facility were most common in CAUTI (46.7%) and least common in cUTI (33.3%).

**Conclusion:**

There are nearly 3 million hospital admissions with a UTI, comprising fully 8% of all annual admissions in the US. Though most are considered uncomplicated, there are few differences in characteristics or outcomes across the categories.

**Disclosures:**

**Marya Zilberberg, MD, MPH**, **Cleveland Clinic** (Consultant)**J&J** (Shareholder)**Lungpacer** (Consultant, Grant/Research Support)**Merck** (Grant/Research Support)**scPharma** (Consultant)**Sedana** (Consultant, Grant/Research Support)**Spero** (Grant/Research Support) **Brian Nathanson, PhD**, **Lungpacer** (Grant/Research Support)**Merck** (Grant/Research Support)**Spero** (Grant/Research Support) **Kate Sulham, MPH**, **Spero Therapeutics** (Consultant)

